# Do patients with well-functioning total hip arthroplasty achieve typical sagittal plane hip kinematics? A proof of concept study

**DOI:** 10.1177/11207000211044471

**Published:** 2021-09-08

**Authors:** Ben Langley, Richard M Page, Chris Whelton, Oliver Chalmers, Stewart C Morrison, Mary Cramp, Paola Dey, Tim N Board

**Affiliations:** 1 Sport and Physical Activity, Faculty of Arts and Sciences, Edge Hill University, Ormskirk, UK; 2 Centre for Hip Surgery, Wrightington Hospital, Wigan Wrightington and Leigh NHS Trust, Wigan, UK; 3 Centre for Doctoral Training in Prosthetics and Orthotics, School of Health and Society, University of Salford, Salford, UK; 4 School of Health Sciences, University of Brighton, Brighton, UK; 5 Allied Health Professions, Health and Applied Sciences, University of the West of England; Bristol, UK; 6 Faculty of Health and Social Care, Edge Hill University, Ormskirk, UK

**Keywords:** Gait, hip surgery, individual responses, motion analysis, walking

## Abstract

**Background:**

Total hip arthroplasty (THA) patients have been shown to not achieve normal sagittal plane hip kinematics. However, previous studies have only conducted group level analysis and as such lack the sensitivity to highlight whether individual patients do achieve normal hip kinematics. As such this study looked to determine whether some patients with well-functioning THA achieve typical sagittal plane hip kinematics.

**Methods:**

Sagittal plane hip kinematics were collected on 11 well-functioning THA patients (Oxford Hip Score = 46 ± 3) and 10 asymptomatic controls using a 3-dimensional motion analysis system during self-paced walking. High-functioning THA patients were identified as those who displayed sagittal plane hip kinematics that were within the variance of the control group on average, and low-functioning patients as those who did not.

**Results:**

5 THA patients were identified as high-functioning, displaying hip kinematics within the variance of the control group. High-functioning THA patients displayed peak hip flexion and extension values more closely aligned to asymptomatic control group than low-functioning patients. However, hip range of motion was comparable between high- and low-functioning total hip arthroplasty patients and reduced compared to controls.

**Conclusion:**

The presence of high-functioning THA patients who display comparable sagittal plane hip kinematics to controls suggests these patients do achieve normative function and challenges the conclusions of previous group level analysis. Understanding why some patients achieve better function post-operatively will aid pre- and post-operative practices to maximise functional recovery.

## Introduction

Total hip arthroplasty (THA) is a common surgical procedure which is used to alleviate pain and enhance function in people with osteoarthritis, after conservative treatments have failed.^1,2^ THA is associated with excellent clinical outcomes, with patients reporting reduced pain and enhanced quality of life.^3^ Despite this, studies utilising 3-dimensional gait analysis have typically concluded that THA patients still display altered hip kinematics postoperative when compared to asymptomatic controls.^4–8^ Specifically, hip extension, adduction, sagittal and frontal plane range of motion (ROM) have been reported to be lower within THA populations compared to controls.^4–8^

Previous studies exploring the extent to which patients achieve normative hip kinematics post-THA have traditionally focused on group level comparisons between THA populations and asymptomatic controls.^4–8^ While this study design enables the extrapolation of findings to the wider population being assessed, it is unable to identify if individuals within the THA group(s) do achieve normative hip function during walking gait, even if the group do not. Exploration of the data on an individual level would provide a means of identifying if individuals do achieve normative walking patterns. Identification of individuals who do and do not (assuming some do) achieve normative walking patterns would enable the creation of functional groups of high- and low-functioning THA patients. Identification of these functional groups would aid the elucidation of factors that influence the magnitude of recovery post-THA with a view to maximising functional recovery postoperatively.

Comparison of the standard deviations (SD) reported for asymptomatic and THA populations in previous studies demonstrates that THA groups are less homogenous than their asymptomatic counterparts.^5,7^ For example, the SDs reported by Bennett et al.^5^ and Varin et al.^7^ for their THA groups are double those reported for the control groups, in relation to both hip extension and ROM. The increased variance evident within the THA population supports the notion that sub-groups of high-functioning (HF) and low-functioning (LF) patients may exist. As such, the aim of this proof of concept study was to compare sagittal plane hip kinematics between THA patients with perceived well-functioning implants and asymptomatic controls, and then to explore whether sub-groups of HF and LF patients were evident within the THA population. Within this context a HF patient may be defined as someone who achieves sagittal plane hip kinematics within the SD of the control group, and LF as a patient who does not. The hypotheses tested within this study were that the THA group would display reduced sagittal plane hip kinematics compared to the asymptomatic controls, but that a sub-set of HF patients would be evident within the THA population.

## Methods

### Participants

11 THA patients and 10 healthy controls were recruited for this study (Table 1), based upon sample size calculations undertaken using G*Power 3.1 and hip extension and sagittal plane ROM data from Varin et al.^7^ and Bennett et al.^5^, Faul et al.^9^ Inclusion criteria for the control group specified that the participants had no known medical conditions which may influence gait. Inclusion criteria for the THA group were: unilateral THA for osteoarthritis at least 1 year prior to recruitment (mean ± SD time postoperative; 22 ± 16 months [minimum; 12 months, maximum; 62 months]); no evidence of loosening of THA on x-ray; body mass index <40 kg/m^2^, with no other known pathologies; arthroplasty or neurological conditions known to influence gait; and able to walk 10 metres unaided. THA participants within this study underwent a posterior surgical approach and perceived their implants to be well-functioning, reporting, on average, excellent Oxford Hip Scores (mean ± SD; 46 ± 3). All participants provided written informed consent and ethical approval for the study was granted by the National Health Research Authority (17/LO/1584).

**Table 1.** Descriptive characteristics for the asymptomatic control and total hip arthroplasty (THA) groups. Mean (standard deviation).

	Control	THA	*p*	*g*
**Age (years)**	**61 (5)**	**71 (8)**	**0.002**	**−1.30**
Height (m)	1.66 (0.09)	1.64 (0.13)	0.675	0.15
Mass (kg)	70 (13)	78 (22)	0.418	−0.30
BMI (kg/m^2^)	25 (3)	29 (5)	0.115	−0.56
Male:Female	4:6	7:4	0.290^a^	–
Walking velocity (m/sec)	1.3 (0.1)	1.1 (0.3)	0.096	0.67

Note: Significant differences are identified in bold font.

g, Hedge’s g; BMI, body mass index.

a Value from Mann-Whitney U-test.

### Procedures

The study was undertaken within a laboratory setting between February 2018 and August 2019. Participants attended a single testing session, in which they were asked to walk at a self-selected velocity along a 7 m walkway until 7 valid trials were recorded. Valid trials were those in which the participant landed on the force plate(s) without any noticeable deviations in their movement pattern. Walking velocity was monitored using 2 single beam timing gates (SmartSpeed, Fusion Sport, Brisbane, Australia) set at torso height, with only trials that were within 5% of the participant’s mean walking velocity accepted during data processing. Kinematic data were collected using a 10-camera motion capture system (Oqus 3+, Qualisys, Gothenburg, Sweden), sampling at 200 Hz. 2 force plates (Kistler, Winterthur, Switzerland), sampling at 2000Hz, imbedded in the centre of the walkway and synchronised with the motion capture system recorded ground reaction forces. A short static trial was collected with the participant stood in a relaxed position to enable the relevant segmental co-ordinate systems to be calculated prior to dynamic data capture.

A 7 segment, 6° of freedom model which defined the pelvis, thighs, shanks and feet was used to calculate hip, knee and ankle joint kinematics. However, only hip joint data were reported within this study. To model the pelvis and thigh 9 mm diameter retro-reflective markers were attached to both limbs using double-sided tape on the anterior and posterior superior iliac spines, and the medial and lateral femoral epicondyles. Additionally, rigid tracking clusters consisting of 4 non-colinear markers were attached to the distal-lateral aspect of the thighs, in line with the calibrated anatomical system technique.^10^ The hip joint centre was calculated using regression equations developed by Bell et al.^11^ Segmental coordinate systems were oriented as follows; x = medial-lateral, y = anterior-posterior and z = vertical. Hip joint data from the operated limb only for the THA group and data for an arbitrarily selected limb for the healthy group is presented.

### Data processing

Marker trajectories were reconstructed and labelled within Qualisys Track Manager (Version 2.18.1, Qualisys, Gothenburg, Sweden) and exported as C3D files to Visual 3D. 5 valid trials per participant were extracted for analysis. Hip joint angles and moments, in all 3 anatomical planes, were calculated using an XYZ cardan sequence of rotations. Kinematic data were filtered using a 6 Hz Butterworth low pass filter and were time normalised to 100% gait cycle duration. Gait cycle events were calculated using the automatic gait events option in Visual 3D, from the vertical ground reaction force data (10 N threshold) and the position of the relevant foot segment. Peak hip flexion, extension and sagittal plane ROM were extracted for analysis. Analysis was limited to these variables based upon the parameters used within the sample size calculation to ensure sufficient statistical power was achieved.

### Data analysis

Data analysis was undertaken in 2 phases. Initially, independent samples *t-*tests were used to compare discrete parameters between groups (THA and control), after confirming all variables met parametric assumptions. The alpha level was set a *p* ⩽ 0.05 and no corrections for multiple comparisons were made in line with the recommendations of Prescott.^12^ Effect sizes were calculated using Hedge’s g with corrections applied to account for the small sample size,^13^ and interpreted as follows; trivial < 0.20, small 0.20–0.49, medium 0.50–0.79 or large ⩾0.80.^14^ All data analysis was undertaken using SPSS Statistics 23 (IBM, New York, USA).

HF and LF THA patients were identified based upon sagittal plane hip kinematics. For each THA patient the absolute difference between their sagittal plane hip angle and the group mean hip angle for the control group was calculated on a point-by-point basis over the walking gait cycle. THA patients whose average difference from the control group over the walking gait cycle was below 1 SD (5.4°) of the control groups mean value were deemed to be HF. Those above this threshold were classified as LF. This approach of using one standard deviation from the control group mean to classify HF and LF groups is consistent with the work of De Pieri et al.,^15^ who classified THA patients based upon walking velocity. Once THA patients were classified as HF or LF the following variables were calculated and compared: mean difference over the walking gait cycle; difference in peak hip flexion; extension; and ROM relative to the control group. No inferential statistical analysis was undertaken once THA participants were sub-grouped due to the small sample size. However, effect sizes were calculated, again using Hedge’s *g*, to estimate the potential impact of differences in the magnitude of the change from the asymptomatic control group between HF and LF THA groups.

## Result

### Comparison of control and THA groups

**Figure 1.**
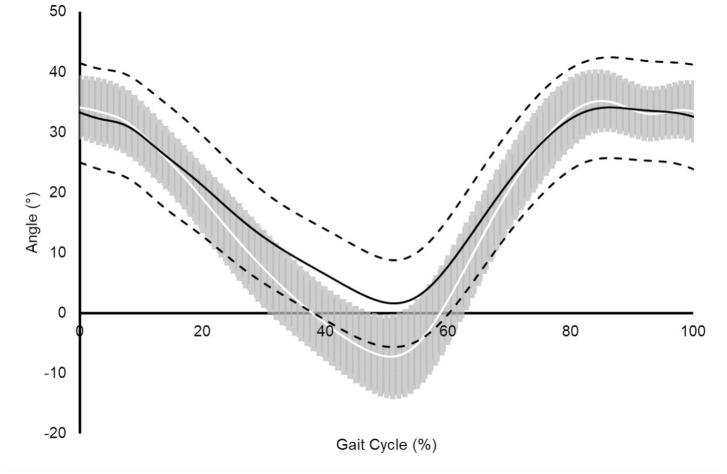
Assembled average sagittal plane hip angle time-history during the walking gait cycle for asymptomatic control group (solid white line; *n* = 10), pooled THA group (solid black line; *n* = 11), respectively. SD are shown as shaded grey region for the asymptomatic control and dashed black lines for the THA groups. Positive values represent hip flexion and negative values hip extension.

THA patients displayed significant (*p* ⩽ 0.008) reductions of 8.6° and 10.0° in peak hip extension and sagittal plane ROM, respectively, compared to the control group, with both changes associated with large effect sizes (*g* > 0.80) (Table 2) (Figure 1). No significant (*p* = 0.617) difference and a small effect size (*g* = 0.18) was reported for peak hip flexion between groups.

**Table 2.** Comparison of selected sagittal plane hip kinematic parameters during the walking gait cycle between asymptomatic control and total hip arthroplasty (THA) groups. Mean (standard deviation).

	Control	THA	*p*	*g*
Peak Flexion (°)	36.1 (4.6)	34.6 (8.2)	0.617	0.18
**Peak Extension (°)**	**−7.4 (6.3)**	**1.2 (7.0)**	**0.008**	**1.06**
**Range of Motion (°)**	**43.5 (3.8)**	**33.5 (5.4)**	**<0.001**	**1.72**

Note: Significant differences highlighted in bold font.

### Comparison of control and THA subgroups

**Figure 2.**
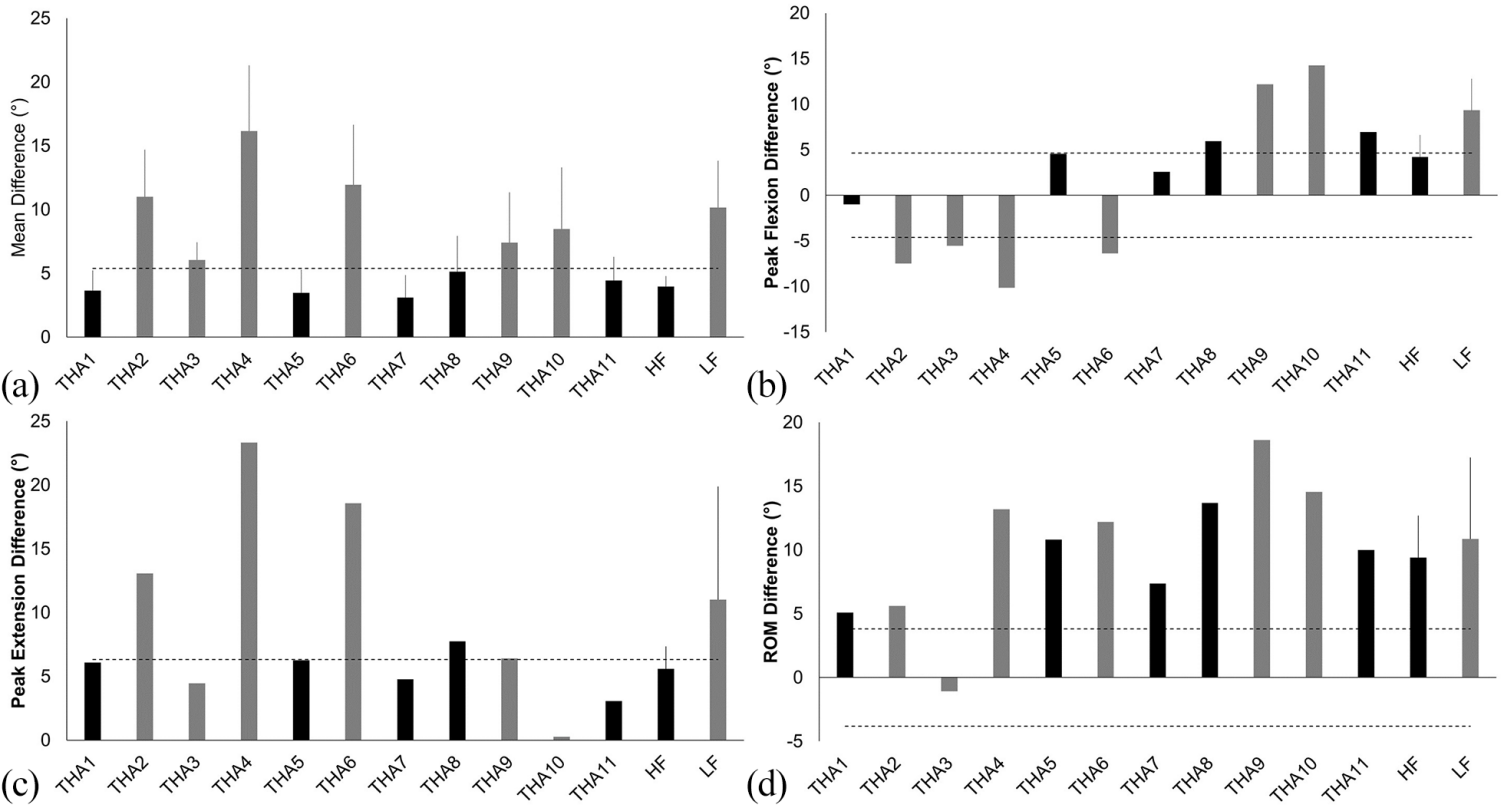
Differences in sagittal plane hip kinematics between THA patients and asymptomatic controls. HF patients are identified in black and LF in grey. Horizontal dashed lines identify the standard deviation reported for the asymptomatic control group. Mean differences between THA and control groups are reported as the average absolute difference. Positive values denote reduced values within the THA populations compared to the asymptomatic controls for flexion, extension and range of motion.

5 THA participants were identified as HF, displaying sagittal plane hip kinematics within 1 SD of the control group over the walking gait cycle, with the remaining 6 THA patients identified as LF (Figure 2(a)). Descriptive characteristics for the HF and LF THA sub-groups are displayed in Table 3. HF patients were on average shorter, lighter and had lower BMI than the LF group with large effect sizes reported for the differences in body mass and BMI, and a medium effect size for the difference in height. Additionally, a greater proportion of females were classified as HF (3/4), whereas a smaller proportion of males were classified as HF (2/7). Age, walking velocity and postoperative Oxford Hip Scores were similar between groups, with differences in these variables associated with small effect sizes.

**Table 3.** Descriptive characteristics for the HF and LF THA sub-groups. Mean (standard deviation).

	HF	LF	*g*
Age (years)	73 (9)	70 (7)	0.28
Height (m)	1.58 (0.10)	1.69 (0.14)	−0.74
Mass (kg)	63 (9)	88 (22)	−1.19
BMI (kg/m^2^)	25 (4)	31 (5)	−0.93
Male:Female	2:3	5:1	–
Walking velocity (m/sec)	1.2 (0.3)	1.1 (0.3)	0.25
Oxford Hip Score	45 (5)	47 (1)	−0.14

HF, high-functioning; LF, low-functioning; *g*, Hedge’s g; BMI, body mass index.

**Figure 3.**
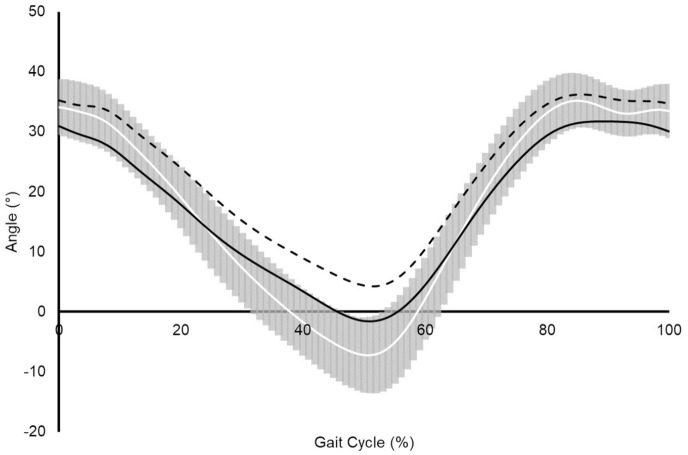
Assembled average sagittal plane hip kinematics during the walking gait cycle for asymptomatic control group (solid white line and grey SD bars; *n* = 10), HF (short dashed black line; *n* = 5) and LF (long dashed black line; *n* = 6) THA sub-groups.

Unsurprisingly, given the classification method, the LF group displayed sagittal plane hip kinematics that were less comparable to the control group than the HF group (Figures 2(a) and 3). On average the mean difference in sagittal plane hip kinematics was 6.2° larger for the LF patients compared to the HF patients, a difference associated with a large effect size (*g* = 1.84). 3 HF patients displayed peak hip flexion within 1 SD of the asymptomatic control group; in contrast, no patients classified as LF displayed peak hip flexion within 1 SD of the control group (Figure 2(b)). The LF THA patients displayed differences in peak hip flexion 5.1° larger on average than the HF group, with this difference also associated with a large effect size (*g* = 1.39). 4 out of 5 HF patients and 2 out of 6 LF patients achieved peak hip extension within 1 SD of the control group (Figure 2(c)). The difference in peak hip extension for the LF group was again larger than for the HF group, by 5.4° on average and had a medium effect size (*g* = 0.67). No HF patients achieved hip ROM within 1 SD of the asymptomatic cohorts mean value, 1 patient classified as a LF did utilise a ROM within 1 SD of the asymptomatic control group (Figure 2(d)). The magnitude of the reduction in hip ROM reported for each THA sub-group was comparable, with a mean difference of 1.5° between HF and LF sub-groups, which had a small effect size (*g* = 0.23).

## Discussion

The aim of this proof of concept study was to compare sagittal plane hip kinematics between THA patients with perceived well-functioning implants and asymptomatic controls, and then to explore whether sub-groups of HF and LF patients were evident within the THA population. The findings of this study support both hypotheses tested, with group level comparison identifying significant reductions in peak hip extension and RoM in the THA patients, and individual assessments revealing a group of HF THA patients who achieve normative motion patterns. The presence of HF and LF individuals within the THA population highlights the lack of homogeneity within this population, while the kinematic patterns of the HF sub-group challenge the conclusions drawn from previous group level comparisons that THA patients do not achieve “normal” hip kinematics, assuming normal is represented by the motion pattern of the asymptomatic control group.

The findings of the group level comparison undertaken are comparable with previous studies,^4–8^ with the THA group displaying significant reductions in peak hip extension and sagittal plane ROM compared to the asymptomatic control group. Not only are the directions of change the same as previous studies,^4–8^ the magnitude of the reduction in peak hip extension (8.6°) and sagittal plane ROM (10.0°) is within the range of deficits reported by Varin et al.^7^ and Bennett et al.,^5^ who reported peak extension and ROM to be reduced by between 4.3° and 15.5°, and 7.9° and 11.2° respectively. These findings demonstrate that the THA and control populations utilised within this study are representative of those used previously within the literature.^4,5,7^ Furthermore, the group level comparison would lead to the same conclusion as previous studies,^4–8^ that THA patients do not achieve normal hip kinematics.

Exploration of the data on an individual level suggests that THA populations are not homogenous, and the findings of this analysis challenge the conclusion that THA patients do not achieve normal sagittal plane hip kinematics. 5 patients within the THA group achieved motion patterns that were on average within the variance of the asymptomatic control group. Displaying sagittal plane hip kinematic patterns within the variance of the asymptomatic group suggests these HF THA patients achieve what could be considered normative motion patterns. Understanding why some THA patients achieve motion patterns that are more comparable to asymptomatic controls and why others do not would help to develop means of maximising functional recovery, and potentially enhance both patient quality of life and implant survivorship through more normal loading of the implant. A range of factors such as patient characteristics, preoperative gait mechanics, implant and or surgical technique, postoperative rehabilitation and patient engagement within this process, strength and flexibility may contribute to the magnitude of functional recovery post-THA.

Exploration of these variables in HF and LF THA populations may elucidate mechanisms to further enhance functional recovery. While this proof of concept study has neither the study design nor the statistical power to explore the impact of these factors upon hip kinematics within the THA population. There is, however, a trend towards female patients with lower BMI being more likely to be classified as HF, and interestingly these groups have been reported to have a significantly reduced risk of requiring revision to their THA.^16,17^ While, Oxford Hip Scores were comparable between groups suggesting that there may be a disconnect between patient’s perceptions and the extent to which postoperative hip motion returns to normal.

The data generated within this study does however provide some insight into the manner in which the HF THA subgroup achieve motion patterns more closely aligned with the asymptomatic controls, compared to the LF THA subgroup. The HF THA subgroup typically achieved peak hip flexion and extension that was within the variance of the asymptomatic control group. However, despite displaying sagittal plane hip kinematics that were on average within the variance of the asymptomatic control groups mean value the HF THA subgroup did not achieve comparable ROM values. Sagittal plane hip ROM was still smaller within the HF THA group and outside of 1 SD of the asymptomatic control groups mean value. This reduction in ROM exemplifies what is evident visually in Figure 3, with the HF THA subgroup displaying hip flexion and extension within the lower boundaries of the asymptomatic control groups variance.

The findings of this study, as always, need to be interpreted in light of the limitations of the work. Walking velocity differed, albeit not significantly, between the THA and asymptomatic control populations and as such some of the differences in hip kinematics both on a group and individual level are likely to be due to this. While some previous studies have taken steps to experimentally control walking speed, for instance selecting trials closest to 1 m/second,^4,8^ the kinematic patterns reported in this instance may not truly be reflective of the natural patterns utilised by participants. Another limitation of the study was the significant difference in age between the THA and asymptomatic control populations, with the controls being younger. While this is not uncommon, with previous studies highlighting the difficulties in recruiting healthy individuals who have no known conditions which may influence walking gait,^4,5,8^ there is the possibility that some of the differences reported between groups are a function of age. Despite the age difference between the groups HF THA patients were still identified and with more closely matched controls the expectation would be that a larger number of patients would be likely to achieve more normative hip kinematics. Finally, the study was a proof of concept study and only powered to identify differences in sagittal plane hip kinematic parameters. Thus, the sample size is small and may be considered a limitation of the study. As such larger scale studies, potentially beginning with reanalysis of existing data sets, are required to explore whether those who display more normal sagittal plane hip kinematics also have frontal or transverse plane motion patterns or joint moments more aligned to asymptomatic controls.

In conclusion, group level comparisons undertaken support previous work which suggest THA patients do not achieve hip kinematics that are comparable to asymptomatic individuals. However, assessment of the data on an individual level reveals that 5 of the THA participants assessed displayed sagittal plane hip kinematics within the variance of the asymptomatic control group. Thus, they could be suggested to achieve normal joint kinematics. Identifying the factors contributing to whether THA patients are classified as HF or LF to the implant will help to develop more effective (p)rehabilitation programmes, surgical procedures and intervention management programmes with a view of maximising functional recovery post-THA.
